# Multi-label classification for multi-drug resistance prediction of *Escherichia coli*

**DOI:** 10.1016/j.csbj.2022.03.007

**Published:** 2022-03-10

**Authors:** Yunxiao Ren, Trinad Chakraborty, Swapnil Doijad, Linda Falgenhauer, Jane Falgenhauer, Alexander Goesmann, Oliver Schwengers, Dominik Heider

**Affiliations:** a Department of Data Science in Biomedicine, Faculty of Mathematics and Computer Science, Philipps-University of Marburg, Germany; b Institute of Medical Microbiology, Justus Liebig University Giessen, Germany; c German Center for Infection Research, Partner site Giessen-Marburg-Langen, Germany; d Institute of Hygiene and Environmental Medicine, Justus Liebig University Giessen, Germany; e Hessisches universitäres Kompetenzzentrum Krankenhaushygiene, Germany; f Department of Bioinformatics and Systems Biology, Justus Liebig University Giessen, Germany

**Keywords:** Multi-drug resistance, Machine learning, Multi-label classification, AMR, Antimicrobial Resistance, MDR, Multi-Drug Resistance, MLC, Multi-Label Classification

## Abstract

Antimicrobial resistance (AMR) is a global health and development threat. In particular, multi-drug resistance (MDR) is increasingly common in pathogenic bacteria. It has become a serious problem to public health, as MDR can lead to the failure of treatment of patients. MDR is typically the result of mutations and the accumulation of multiple resistance genes within a single cell. Machine learning methods have a wide range of applications for AMR prediction. However, these approaches typically focus on single drug resistance prediction and do not incorporate information on accumulating antimicrobial resistance traits over time. Thus, identifying multi-drug resistance simultaneously and rapidly remains an open challenge. In our study, we could demonstrate that multi-label classification (MLC) methods can be used to model multi-drug resistance in pathogens. Importantly, we found the ensemble of classifier chains (ECC) model achieves accurate MDR prediction and outperforms other MLC methods. Thus, our study extends the available tools for MDR prediction and paves the way for improving diagnostics of infections in patients. Furthermore, the MLC methods we introduced here would contribute to reducing the threat of antimicrobial resistance and related deaths in the future by improving the speed and accuracy of the identification of pathogens and resistance.

## Highlights

• Multi-label classification (MLC) methods can be used to reliably predict multi-drug resistance in pathogens.
• ECC model outperforms the other four MLC methods and can effectively predict MDR with high accuracy.
• Our study paves the way for improving diagnostics of infections in patients.

## Graphical abstract

**Figure:**
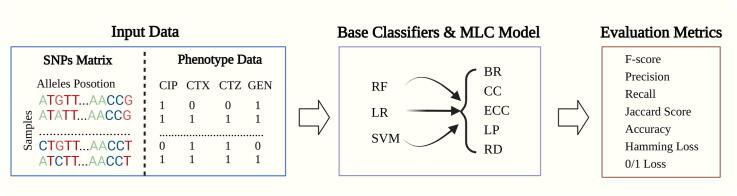

## 1 Introduction

Antimicrobial resistance (AMR) is rapidly increasing and is, therefore, one of the greatest threats to global health and also causes significant economic problems. According to WHO estimates, without countermeasures, up to 10 million deaths will be caused by AMR in the future, with immense costs to the healthcare system of approximately $100 trillion by 2050 [1]. In particular, infection due to multi-drug resistance (MDR) pathogens has become most threatening to public health, as MDR can lead to failure of treatment of patients [2], [3]. For instance, the emergence of MDR in *Escherichia coli (E. coli)* has become one of the global health concerns [4], [5], [6]. In general, bacteria are resistant to antibiotics by spontaneous mutations in existing genes or by the acquisition of extraneous genes [6], [7]. Many previous studies investigating AMR have focused on well-known resistance genes or mutations in well-known genes, such as mutations in the *gyrA* gene and *parC* gene in *E. coli* [8], [9]. However, there is a lack of AMR studies based on overall mutations without previous knowledge.

While antimicrobial susceptibility testing (AST) is widely used for AMR profiles in clinical practice, machine learning models have been shown to produce highly reliable predictions in a shorter turnaround time. Typically, these machine learning models combine sequencing data with antibiotic resistance databases with phenotypic information [10], [11]. For instance, Yang *et al.*, [12] and Kouchaki *et al*., [13] used different machine learning algorithms, namely support vector machine (SVM), logistic regression (LR), and random forest (RF) to predict AMR from whole-genome sequencing data and achieved high accuracy prediction. Other approaches also included deep learning to predict new antibiotic drugs, AMR genes, and AMR peptides [14], [15], [16], [17], [18], [19], [20]. However, all of these studies are based on single drug resistance information and do not take into account the MDR information of the bacteria.

Multi-label classification (MLC) offers a potential solution for AMR prediction based on MDR information. Traditionally, multi-label problems are transformed into single-label problems [21]. For instance, the widely known binary relevance (BR) approach, is a simple and straightforward method that treats each label as an independent binary problem [22]. One of the limitations of the BR approach is that it does not take into account the dependencies between the labels [23]. Unlike BR, the classifier chain (CC) takes into account the correlation among labels and uses the predicted results from the previous classifiers as an additional input for the following classifier [24]. Obviously, the order of the CC affects the prediction accuracy. Thus, the ensemble of classifier chains (ECC) was proposed, which contains several CCs with different orders and can be applied to study the dependencies between labels [23], [24]. CCs and ECCs have been used for cross-resistance prediction in HIV based on protein sequences of the HIV-1 reverse transcriptase [25] and protease [26], however, it has never been used with genomic data and MDR of bacteria. Other multi-label approaches include the label powerset (LP) method, which considers the dependency among labels, and each label combination is considered as a class [21]. Random label space partitioning with label powerset (RD) method is another effective ensemble method, which is based on label powerset with a random subset of *k* labels [23], [24].

In our study, we gave the applications of MLC methods on multi-drug resistance prediction. We aimed at identifying secondary mutations that contribute to the resistance directly or indirectly, e.g., compensatory mutations. We did not include the known resistance genes. Our approach does not need any AMR expert knowledge and can also predict resistance even without knowing the resistance genes by identifying secondary mutations. The results demonstrated that the ECC model can significantly improve overall resistance prediction in bacteria compared to the other four MLC methods. MLC models will improve patient care, in particular the treatment of patients, reduce the threat of antimicrobial resistance and related deaths in the future, and improve the speed and accuracy of the identification of pathogens and resistance.

## 2 Materials and methods

### 2.1 Dataset

In our analysis, we used 987 whole-genome sequencing (WGS) data of *E. coli* strains with resistance information for four antibiotics, namely ciprofloxacin (CIP), cefotaxime (CTX), ceftazidime (CTZ), and gentamicin (GEN). These data were collected by our partner institution, the University of Giessen. The isolates were obtained from human and animal clinical samples. Antimicrobial susceptibility testing was performed using the VITEK® 2 system (bioMérieux, Nürtingen, Germany) and interpreted following EUCAST guidelines. DNA isolation and whole-genome sequencing was performed as described in Falgenhauer et al. [27].

In order to use MLC, the isolates need to be filtered for missing antibiotic resistance information. The final dataset with complete MDR information contains 809 *E. coli* strains (see Table 1). CIP is a fluoroquinolone and is widely used to treat infections with Gram-negative bacteria, e.g., gastroenteritis, respiratory tract infections, or urinary tract infections [28]. CTX and CTZ are broad-spectrum antibiotics from the class of cephalosporins and are widely used to treat infections of Gram-positive and Gram-negative bacteria, such as meningitis, pneumonia, urinary tract infections, sepsis, and gonorrhea [29], [30]. GEN is an aminoglycoside and is widely used to treat various infections of Gram-negative bacteria, including meningitis, pneumonia, urinary tract infections, and sepsis [31].

**Table 1:** Overview of the dataset.

Antibiotics	CIP	CTX	CTZ	GEN
Resistant	366	358	276	188
Susceptible	443	451	533	621

### 2.2 Dataset pre-processing and encoding

The pre-processing step of raw WGS data refer to our previous study [20]. Briefly, we filtered bad quality reads by fastp (v0.23.2) software [32] and then mapped the clean reads to *E. coli* reference genome (*E. coli* K-12 strain. MG1655) through BWA-MEM with default parameters [33]. We called single nucleotide polymorphisms (SNPs) variants using bcftools (v1.14) via ‘call’ function with default parameters [34], [35]. We extracted reference alleles, variant alleles and their positions, and merged all isolates based on the position of reference alleles. We retained the alleles existing variant more than half in samples. Finally, we got an SNP matrix, where the rows represent the samples and columns are the variant alleles. We utilized one-hot encoding to transform the SNP matrix into a binary matrix for subsequent machine learning.

### 2.3 Multi-label classification

**Fig. 1:**
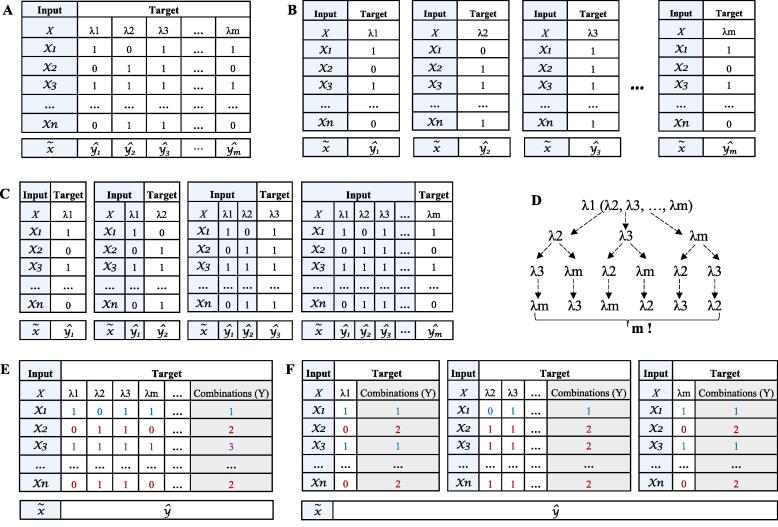
Transformation methods of multi-label classification problems. (A) One multi-label dataset. χ_i_ ∈ xis a training instance. (B) Binary relevance (BR) transforms the multi-label dataset with *m* labels into *m* independent binary datasets. (C) The process of classifier chain (CC) for multi-label data. (D) The possible number of label orders for ensemble classifier chains (ECC). (E) The transformation of the multi-label dataset by label powerset (LP). Labels with different colors represent the different combinations of labels. (F) The transformation of a multi-label dataset by random label space partitioning with label powerset (RD). Labels with different colors represent the different combinations of labels.

In the current study, we used BR, CC, ECC, LP, and RD for the multi-label classification of MDR in bacteria. BR is typically used as a baseline model to compare multi-label classification models. Let $L:=\{\lambda_{1},...,\lambda_{m}\}$ with m>1 be a finite set of class labels (here: resistance for the four antibiotics), and let X be the instance space, i.e., the SNPs. The training set S in MLC is then defined as $S:=\{\left(x_{1},y_{1}\right),...,\left(x_{n},y_{n}\right)\}$, generated independently and identically according to a probability distribution P(X,) on X×Y. Y is the set of possible label combinations, i.e., the powerset of L (Fig. 1A).

BR divides the dataset with *L* labels into *L* binary classification problems (Fig. 1B). Accordingly, we split the data into four binary classification problems, one for each antibiotic (CIP, CTX, CTZ, and GEN). In contrast, the CC approach links the *L* binary classifiers into a “chain” such that the output prediction of one classifier is used as an additional input for all subsequent classifiers, which overcomes the disadvantage of not considering dependencies between labels and captures possible dependencies between the labels (Fig. 1C). The performance of CC depends heavily on the order of the chain, thus, Read *et al*., [23] proposed the use of ECC, which aggregates several chains with different orders by majority vote (Fig. 1D). The LP approach transforms a multi-label problem into a single-label multi-class problem, which is trained on all unique label combinations found in the training data [36] (Fig. 1E). The RD method divides the label space into partitions of size k, trains an LP classifier per partition, and predicts the testing data by aggregating the result of all LP classifiers (Fig. 1F). It is important to note that any standard method for binary classification can be used in these multi-label approaches. In the current study, we evaluated RFs, LR, and SVMs for multi-label classification of MDR in bacteria.

### 2.4 Evaluation metrics

In MLC, the predictions for each instance are a collection of labels, and the performance of classifiers can be calculated through the average score of an evaluation metric or directly by comparing the scores for each class. In this study, we employed seven different metrics that are widely used to evaluate the performance of the classifiers including hamming loss, 0/1 loss, F-score, accuracy, precision, recall, and Jaccard similarity.

The Hamming loss and 0/1 loss are commonly used for the evaluation of MLC models [37]. For Hamming loss, it is defined as the fraction of labels that are incorrectly predicted. The 0/1 loss simply checks whether the complete label subset is predicted correctly or not, represented as the percentage of incorrectly predicted labels.

Accuracy is defined as the proportion of correct predictions, while precision is defined as the number of resistant samples divided by the overall number of samples that are predicted to be resistant. Recall (also called sensitivity) is defined as the number of correctly predicted resistant samples divided by the total number of resistant samples. The F-score can be calculated as the weighted average of precision and recall. Jaccard similarity indicates the overlap between the ground truth and the predictions, focusing on true positives and ignoring true negatives [38]. The classifiers were trained and evaluated based on five-times 5-fold cross-validation, which means the dataset is randomly divided into 5 equal sub-groups, and one of the groups is used as the test set and the rest are used as the training set. The model is trained on the training set and scored on the test set. Then the process is repeated until each unique group has been used as the test set. Statistical significance has been calculated based on the Wilcoxon signed-rank test and T-test.

## 3 Results

### 3.1 Performance of different MLC methods on RF base classifier

**Fig. 2:**
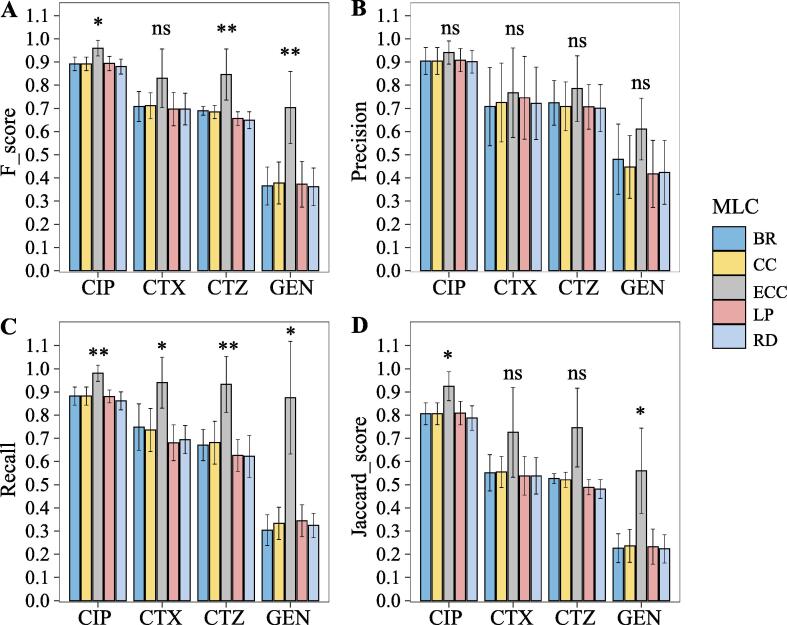
Performance of different MLC methods with RF base classifiers for resistance prediction for each antibiotic. (A) F-scores, (B) Precision, (C) Recall, and (D) Jaccard score of five MLC methods with RF base classifiers for predicting resistance against each antibiotic. ∗ p < 0.05, ∗∗p < 0.01, ∗∗∗p < 0.001, ns: no significance.

We firstly constructed five MLC models (BR, CC, ECC, LP, and RD) based on RF base classifier for MDR prediction of four antibiotics (CIP, CTX, CTZ, and GEN). We compared the performance by F-score, Precision and Recall, and Jaccard score. As shown in Fig. 2, the ECC model has the highest F-score, Precision and Recall, and Jaccard score for resistance prediction against four antibiotics. For instance, the ECC model reached a F-score, precision, recall, and Jaccard score on the CIP dataset of 0.93 ± 0.04, 0.94 ± 0.05, 0.98 ± 0.03, and 0.92 ± 0.06, respectively. Especially, the ECC model significantly outperformed the BR, CC, LP, and RD for predicting resistance against CIP, CTZ, and GEN based on the F-score metric. Moreover, we observed from the Recall metric that the performance of the ECC model is significantly better than other models, which represents the ECC model has a better sensitivity to detect resistant samples. Besides, the ECC model reached, in general, the highest accuracy, as well as, lowest hamming loss, and 0/1 loss for RF (Table 2). Taken together, our results indicated that the ECC models can significantly improve the prediction performance for MDR prediction in *E. coli*.

**Table 2:** Accuracy, hamming loss, and 0/1 loss of five MLC methods with RF base classifier for predicting resistance against four antibiotics. Mean ± standard deviations (significance label of p-value) are shown in table. The statistical significances were compared each group to all (base-mean). ∗p < 0.05, ∗∗p < 0.01, ∗∗∗p < 0.001, ns: no significance.

MLC	Accuracy	Hamming Loss	0/1 Loss
BR	0.51 ± 0.07 (ns)	0.20 ± 0.03 (ns)	0.49 ± 0.07 (ns)
CC	0.52 ± 0.07 (ns)	0.20 ± 0.04 (ns)	0.48 ± 0.06 (ns)
ECC	0.72 ± 0.13 (ns)	0.11 ± 0.05 (*)	0.28 ± 0.13 (ns)
LP	0.53 ± 0.08 (ns)	0.11 ± 0.05 (ns)	0.47 ± 0.08 (ns)
RD	0.51 ± 0.09 (ns)	0.21 ± 0.04 (ns)	0.49 ± 0.09 (ns)

### 3.2 Performance of different MLC methods on LR base classifier

**Fig. 3:**
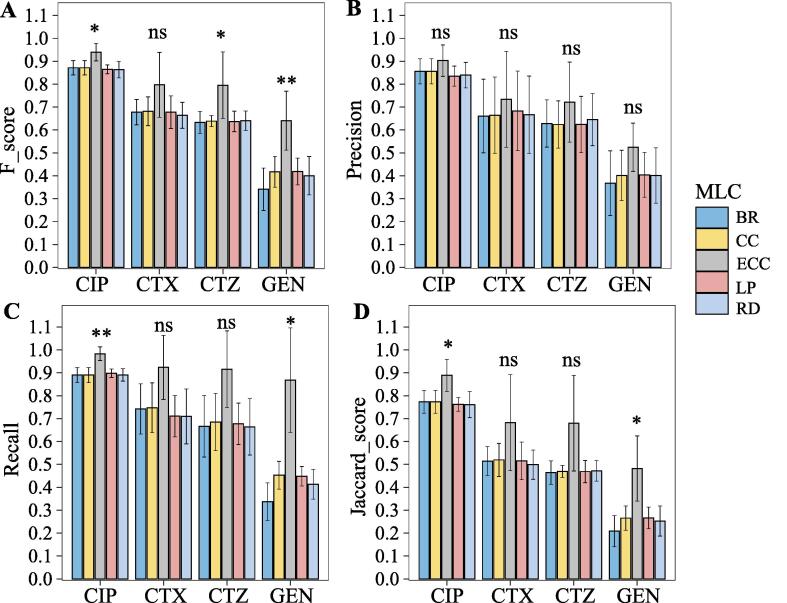
Performance of different MLC methods with LR base classifiers for resistance prediction for each antibiotic. (A) F-scores, (B) Precision, (C) Recall, and (D) Jaccard score of five MLC methods with RF base classifiers for predicting resistance against each antibiotic. ∗p < 0.05, ∗∗p < 0.01, ∗∗∗p < 0.001, ns: no significance.

We also compared the performance of the five MLC methods (BR, CC, ECC, LP, and RD) on the LR base classifier. We found the ECC model still got a higher F-score, precision, recall, and Jaccard score (Fig. 3), which showed the consistent performance of the ECC model on LR with RF base classifier. The results on F-score suggested that ECC model is significantly better than other models for CIP, CTZ, and GEN drug, reached 0.94 ± 0.04, 0.80 ± 0.15, and 0.64 ± 0.13 (p-value < 0.05). We also found a similar trend in recall results of the ECC model, and the ECC model achieved a higher sensitivity performance for MDR prediction. Moreover, ECC model significantly outperformed other four MLC methods on CIP and GEN drug based on recall results (0.98 ± 0.03, 0.87 ± 0.23, p-value < 0.05) and Jaccard score (0.89 ± 0.07, 0.48 ± 0.14, p-value < 0.05). As well, the ECC model got the highest accuracy, lowest hamming loss, and 0/1 loss on the LR base classifier (Table 3). These results demonstrated that the ECC model still has robust performance for MDR prediction.

**Table 3:** Accuracy, hamming loss, and 0/1 loss of five MLC methods with LR base classifier for predicting resistance against four antibiotics. Mean ± standard deviations (significance label of p-value) are shown in table. The statistical significances were compared each group to all (base-mean). ∗p < 0.05, ∗∗p < 0.01, ∗∗∗p < 0.001, ns: no significance.

MLC	Accuracy	Hamming Loss	0/1 Loss
BR	0.45 ± 0.08 (ns)	0.24 ± 0.04 (ns)	0.55 ± 0.08 (ns)
CC	0.47 ± 0.08 (ns)	0.23 ± 0.04 (ns)	0.53 ± 0.08 (ns)
ECC	0.65 ± 0.11 (ns)	0.14 ± 0.05 (*)	0.35 ± 0.11 (ns)
LP	0.50 ± 0.08 (ns)	0.23 ± 0.04 (ns)	0.50 ± 0.08 (ns)
RD	0.47 ± 0.07 (ns)	0.24 ± 0.05 (ns)	0.53 ± 0.07 (ns)

### 3.3 Performance of different MLC methods on SVM base classifier

**Fig. 4:**
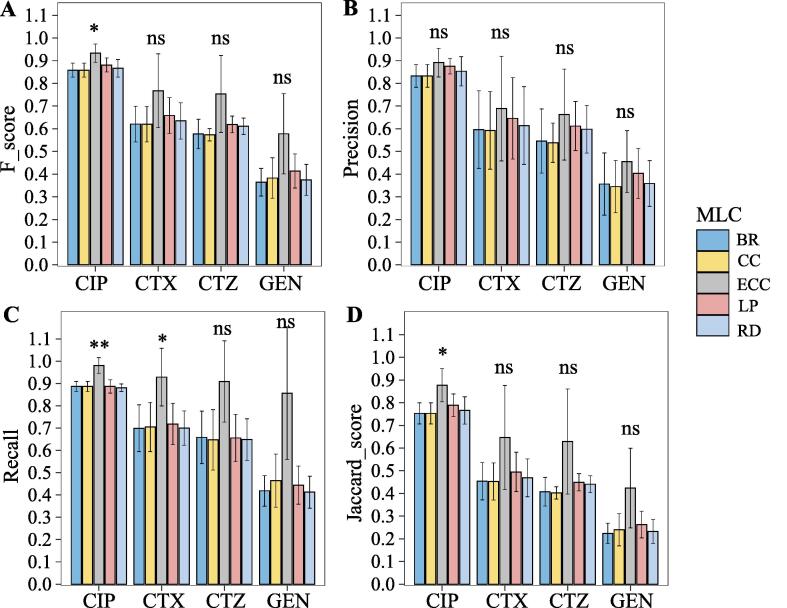
Performance of different MLC methods with SVM base classifiers for resistance prediction for each antibiotic. (A) F-scores, (B) Precision, (C) Recall, and (D) Jaccard score of five MLC methods with RF base classifiers for predicting resistance against each antibiotic. ∗p < 0.05, ∗∗p < 0.01, ∗∗∗p < 0.001, ns: no significance.

For SVM, the F-score of ECC model is significantly better than BR, CC, LP, and RD only for CIP (Fig. 4A) (F-scores of 0.93 ± 0.04, 0.86 ± 0.03, 0.86 ± 0.03, 0.88 ± 0.03, and 0.87 ± 0.04, respectively). There are, however, no significant differences between BR, CC, LP, and RD models. In comparison, CC, LP, and RD did not improve the precision or recall significantly, and in some cases even performed worse compared to the BR (Fig. 4B-C). For the CCs, this might be due to the known problem of error propagation [39]. We found the same conclusion from Jaccard score that the ECC model got better performance than the other four MLC methods, and the Jaccard score of the ECC ranged from 0.42 ± 0.18 for the drug GEN to 0.88 ± 0.07 for the drug CIP (Fig. 4D). Moreover, the ECC model based on the SVM base classifier reached consistent performance with the highest accuracy, lowest hamming loss, and 0/1 loss for RF (Table 4). In summary, the results based on the SVM classifier also demonstrated that the ECC models can significantly improve the prediction performance for MDR prediction in *E. coli*.

**Table 4:** Accuracy, hamming loss, and 0/1 loss of five MLC methods with SVM base classifier for predicting resistance against four antibiotics. Mean ± standard deviations (significance label of p-value) are shown in table. The statistical significances were compared each group to all (base-mean). ∗p < 0.05, ∗∗p < 0.01, ∗∗∗p < 0.001, ns: no significance.

MLC	Accuracy	Hamming Loss	0/1 Loss
BR	0.37 ± 0.08 (ns)	0.28 ± 0.05 (ns)	0.63 ± 0.08 (ns)
CC	0.39 ± 0.08 (ns)	0.28 ± 0.05 (ns)	0.61 ± 0.08 (ns)
ECC	0.57 ± 0.12 (ns)	0.18 ± 0.07 (ns)	0.43 ± 0.12 (ns)
LP	0.47 ± 0.07 (ns)	0.24 ± 0.03 (ns)	0.53 ± 0.07 (ns)
RD	0.41 ± 0.09 (ns)	0.26 ± 0.05 (ns)	0.59 ± 0.09 (ns)

## 4 Discussion

In our study, we compared five MLC models (BR, CC, ECC, LP, and RD) based on three base classifiers (RF, LR, and SVM) for MDR predictions in *E. coli* and evaluated the performance with seven different metrics. Our results illustrated that the ECC model outperforms the other MLC methods and can effectively predict MDR.

The ECC multi-label classification model has a wide range of applications, e.g., for cancers, chronic diseases, and viruses. For instance, Zhou *et al*., [40] reported that the ECC performed best in the diagnosis of four diabetic complications. ECCs have also been used for cross-resistance prediction in viral infections, e.g., in HIV-1 [25], [26]. Here, we firstly applied ECC models on multi-label drug resistance prediction based on all mutations, which could contribute to improving the MDR prediction in other model organisms or poorly known organisms.

Our results also showed that ECC obtained the highest accuracy in all three base classifiers compared to the other four MLC methods, which indicates that the ECC model has good scalability, and can be combined with multiple base classifiers, such as neural networks. Among them, the ECC model based on RF base classifier performs best compared to LR and SVM, which is consistent with our previous research results [20].

The performance of five MLC methods on each drug is different. In general, all MLC methods performed well on CIP drug, and worse on GEN drug. The comparatively lower performance for GEN may be based on the fact that bacterial resistance to GEN is predominantly mediated by plasmids carrying the resistance genes. We focused here solely on chromosomal sequences of the bacteria and did not take into account the effect of alterations in other genetic components on the MDR, like the plasmids, transposons, and integrons [41], [42]. This is one of the limitations of our study. The other limitation in our study is our MLC models are built only on four drugs, and we should integrate more types of antibiotics to further investigate the MDR prediction in the future.

## 5 Conclusions

In summary, our study illustrates five MLC methods based on three base classifiers that achieved accurate MDR prediction. Our results suggest ECC is a promising MLC method for MDR identification, which could be used as a reference approach for clinical staff to improve the diagnostics and patient treatments and thus contribute to reducing the threat of antimicrobial resistance and related deaths in the future.

## Declaration of Competing Interest

The authors declare that they have no known competing financial interests or personal relationships that could have appeared to influence the work reported in this paper.

## Data Availability

Source codes for data preparation and model training are provided at Github website https://github.com/YunxiaoRen/Multi_Label-Classification. And the final SNP matrix datasets we used for model training in this paper are also available at https://github.com/YunxiaoRen/Multi_Label-Classification.
